# Dissecting and Circumventing the Requirement for RAM in CSL-Dependent Notch Signaling

**DOI:** 10.1371/journal.pone.0039093

**Published:** 2012-08-02

**Authors:** Scott E. Johnson, Douglas Barrick

**Affiliations:** T.C. Jenkins Department of Biophysics, The Johns Hopkins University, Baltimore, Maryland, United States of America; University of Sheffield, United Kingdom

## Abstract

The Notch signaling pathway is an intercellular communication network vital to metazoan development. Notch activation leads to the nuclear localization of the intracellular portion (NICD) of the Notch receptor. Once in the nucleus, NICD binds the transcription factor CSL through a bivalent interaction involving the high-affinity RAM region and the lower affinity ANK domain, converting CSL from a transcriptionally-repressed to an active state. This interaction is believed to directly displace co-repressor proteins from CSL and recruit co-activator proteins. Here we investigate the consequences of this bivalent organization in converting CSL from the repressed to active form. One proposed function of RAM is to promote the weak ANK:CSL interaction; thus, fusion of CSL-ANK should bypass this function of RAM. We find that a CSL-ANK fusion protein is transcriptionally active in reporter assays, but that the addition of RAM *in trans* further increases transcriptional activity, suggesting another role of RAM in activation. A single F235L point substitution, which disrupts co-repressor binding to CSL, renders the CSL-ANK fusion fully active and refractory to further stimulation by RAM *in trans*. These results suggest that in the context of a mammalian CSL-ANK fusion protein, the main role of RAM is to displace co-repressor proteins from CSL.

## Introduction

The Notch signaling pathway is a highly conserved, cell-cell communication network through which adjacent cells interact, giving rise to cell differentiation during metazoan development, and stem cell homeostasis in the adult organism. Misregulation of the pathway can lead to devastating effects; e.g., hypoactive Notch signaling can lead to gross tissue malformations during early development, and hyperactive Notch signaling can lead to T-cell acute lymphoblastic leukemia in children [1]–[4].

At the heart of the Notch signaling pathway is the Notch receptor, a 300-kDa single-pass transmembrane receptor protein localized in the plasma membrane. The Notch intracellular domain (NICD) comprises a membrane-proximal RAM (RBP-Jk-associated-molecule) region, a seven ankyrin repeat domain (ANK), a bi-partite nuclear localization sequence (NLS), and a C-terminal PEST degradation motif [5]. The signaling pathway is initiated when a ligand from the DSL (Delta, Serrate, Lag-2, for the mammalian, *D. melanogaster*, and *C. elegans* orthologs, respectively) family on the surface of a neighboring cell binds to the extracellular portion of the Notch receptor. Receptor ligation activates two proteolytic cleavages near the transmembrane region of the Notch receptor. As a result of these cleavages, NICD is released from the plasma membrane and translocates to the nucleus, where it activates transcription of Notch-target genes.

NICD engages the CSL transcription factor through a unique bivalent interaction involving the RAM region and the ANK domain. The RAM and ANK segments are separated by ∼80 residues that are poorly conserved in sequence identity. Biochemical and molecular genetic studies indicate that both the RAM and ANK region are critical for activation [6]. Point substitutions in a conserved xWxP motif in the RAM segment abrogate transcriptional activation [7], as do point substitutions that disrupt the folding of the ANK domain [8]–[10]. Moreover, expression of RAM and ANK separately fail to activate transcription in vertebrates [11]–[12], although in *C. elegans*, the ANK domain alone activates the pathway [13], [14].

**Figure 1:**
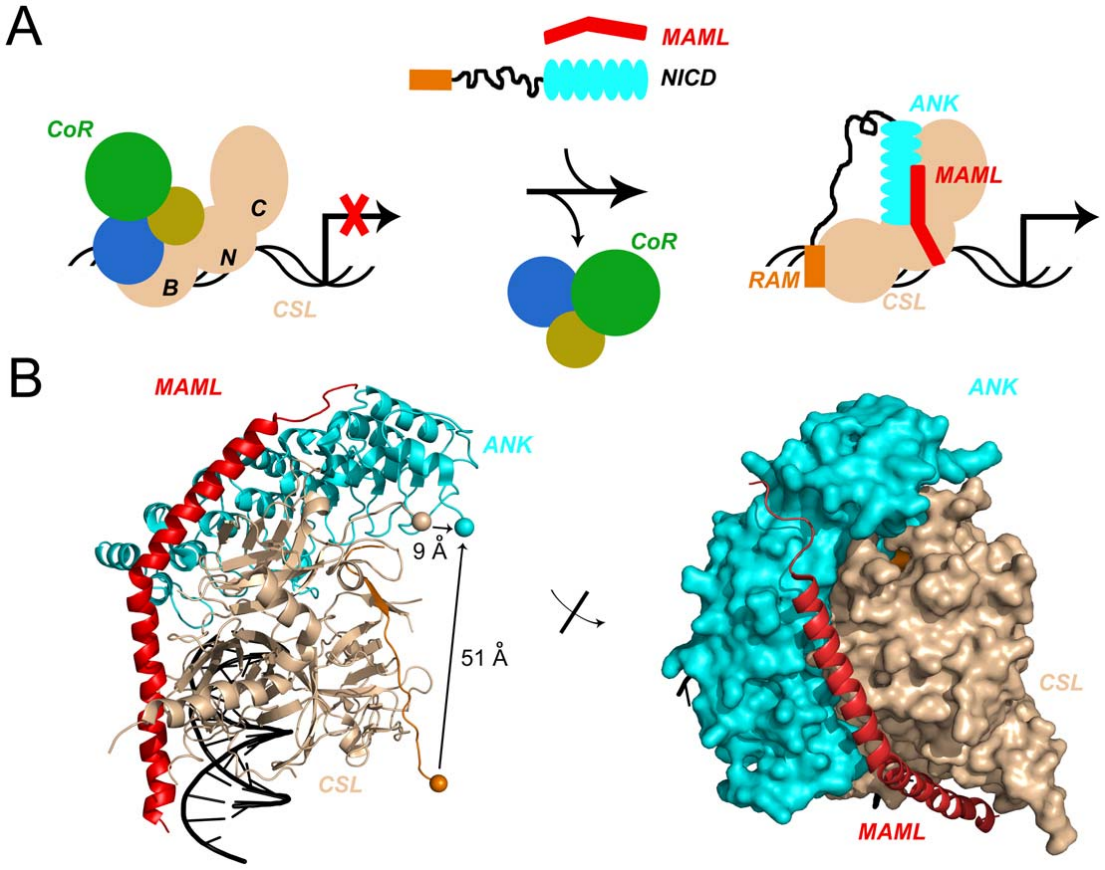
Switching on/off Notch signaling through CSL:NICD interaction. A). In the absence of active Notch signaling, the three-domain transcription factor CSL acts as a transcriptional repressor through interactions with a variety of co-repressor proteins and HDAC. The three domains of CSL are abbreviated N, B, and C for the NTD, BTD, and CTD, respectively. Bivalent binding between NICD and CSL (BTD:RAM and CTD:ANK) is believed to be coupled to co-repressor displacement, allowing for the recruitment of the co-activator protein MAML, resulting in active transcription of downstream target genes. B). Left, Ribbon diagram of the active ternary complex. The arrow highlights a separation of 51 Å between the C-terminus of RAM (orange sphere) and the N-terminus of ANK (cyan sphere). In the crystal structure, the C-terminus of CSL (wheat sphere) and the N-terminus of ANK (cyan sphere) are separated by 9 Å. Right, Surface representation of the active ternary complex (CSL:NICD:MAML; PDB code 2FO1) shows MAML (red) binding at the interface between the CTD of CSL (wheat) and the ANK domain (cyan) of NICD. Molecular representations were generated using PyMOL [49].

Structural studies show that the linker separating RAM and ANK is disordered both in solution [15], [16] and in a crystal structure of a complex including the RAM-ANK segment and CSL [17]. The RAM and ANK binding sites on CSL are distant from one another: RAM binds to the beta-trefoil domain (BTD) of CSL, whereas ANK binds primarily to the C-terminal domain (CTD) of CSL (Figure 1A). The C-terminus of BTD-bound RAM is separated from the N-terminus of CTD-bound ANK by 51 Å in the worm ternary complex crystal structure (Figure 1B).

Given the unique bivalent architecture of the NICD:CSL interaction, what are the mechanistic features of each interaction that contribute to Notch signaling, and how do these two interactions affect one another over this distance? Structural studies show that the ANK:CSL surface provides the primary site for binding of Mastermind-like (MAML), a downstream activator of transcription (Figure 1B) [17], [18]. However, it is the RAM region that contributes the bulk of the affinity to the NICD:CSL complex (K_d_ on the order of 100 nM) [7], [15], [19]–[21], based on i.) studies of binding of RAM fragments to CSL and to the isolated BTD, ii.) the observations that the ANK domain only binds at very high concentrations and requires stabilization by MAML [15], [20], [22], and iii.) in the absence of MAML, RAM binds to CSL with similar affinity as RAMANK [23], differing by two-fold at most [24]. It has been proposed that the covalent linkage of RAM and ANK by an intrinsically disordered segment increases the effective concentration of ANK near CSL, thereby promoting MAML association and downstream activation [16], [25]. Thus, the linker could serve to propagate (in a statistical sense) the high affinity interaction of RAM to enhance ANK:MAML binding to CSL, as has been observed in other model systems [26].

Another proposed mechanism for RAM-mediated activation involves allosteric changes in CSL that positively couple binding of RAM to binding of ANK and/or MAML. Such changes include rigid-body shifts in the domains of CSL [17], as well as more subtle loop rearrangements that may promote MAML binding [20] and as a result, stabilize the CSL:ANK interface. Alignment of worm CSL:DNA and RAM:CSL:DNA crystal structures, though in different space groups, revealed two possible structural rearrangements in CSL associated with RAM binding. One of these conformational changes is a modest rearrangement in the NTD, distal to the site of RAM binding, and has been implicated as a requirement to allow binding of the co-activator MAML in the absence of steric hindrance (Figure S1) [20]. However, these two proposed functions of RAM, to confer an increase in effective concentration of ANK at the CTD of CSL, and to induce conformational changes in CSL, are not mutually exclusive, and can be compatible in a model where RAM promotes the ANK:CTD interaction while inducing rearrangement of the NTD loop to make room for binding of MAML.

In addition to the two proposed functions of RAM discussed above, RAM has been presumed to displace co-repressor proteins from CSL. Numerous co-repressor proteins appear to modulate Notch signaling, namely Hairless, SHARP (MINT), KyoT2, NCor2 (SMRT), CIR, ETO, and MTG16 [27]–[34]. Of these co-repressors, direct competition with NICD binding to CSL has been demonstrated for NCor2, SHARP/MINT, KyoT2, and MTG16 [28], [29], [31], [32], [34]. Early studies on co-repressor proteins identified putative binding sites within BTD (which was then referred to as the ‘repression domain’); since BTD is also the primary (high-affinity) binding site of NICD, the RAM region of NICD was proposed to directly compete with (and displace) the co-repressor proteins from BTD. However, recent biophysical characterization of the MINT:CSL and Hairless:CSL interactions have revealed that MINT binds to both BTD and CTD [23], and that Hairless binds exclusively to CTD [35]. These studies reveal that co-repressors bind domains of CSL other than BTD, and call into question the generalization that all co-repressors are directly displaced by competition with RAM at a single site on BTD.

Given the importance of NICD-mediated switching of CSL from transcriptional repression to activation, we sought to determine the mechanism(s) by which the RAM and ANK regions of NICD contribute, individually and collectively, to Notch signaling through CSL. To test the extent to which RAM activates transcription by increasing the effective concentration of ANK near CSL, we fused the ANK domain of NICD to the C-terminus of CSL *in cis*. As this construct lacks the high affinity RAM sequence, the degree to which this fusion actives transcription should reflect the relative importance of the effective concentration mechanism, as opposed to co-repressor displacement and/or allosteric coupling. The contribution of these alternative mechanisms can be tested by adding RAM to the CSL-ANK fusion *in trans*. Using a CSL-ANK fusion protein, in combination with CSL substitutions that disrupt co-repressor binding, we can begin to resolve the relative contributions of co-repressor displacement and allostery to Notch signaling.

## Results

### Enhancement of the activities of RAM and ANK by covalent linkage in NICD

**Figure 2:**
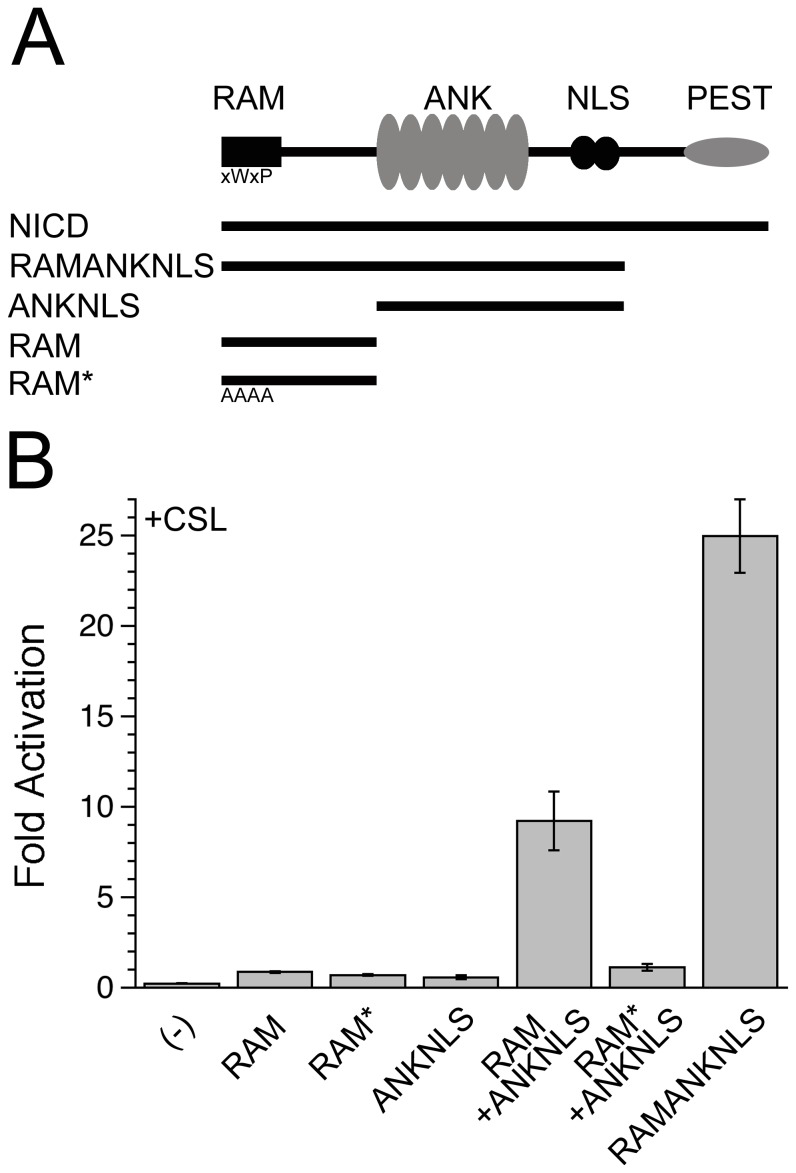
RAM and ANK are both required for transcriptional activation, and require covalent linkage for maximal activation. A). A schematic of the regions comprising NICD, and the constructs used to dissect the function associated with each region. B). Notch-mediated transcriptional activation of NICD terminal truncations in OT11 cells, measured using a luciferase reporter downstream of multiple CSL-binding sites. Although very little activity is detected with RAM alone or ANKNLS alone, substantial activity is obtained when RAM and ANKNLS are co-transfected. Further activation is obtained when RAM and ANKNLS are connected *in cis* by their linker, consistent with an increase in the effective concentration. No activity is observed for the co-transfection of ANKNLS with RAM* (CSL-binding incompetent).

By separating RAM from ANKNLS, we can directly test the extent to which RAM confers an increased concentration of ANK at its binding site on CTD, thus promoting this otherwise weak interaction. Neither ANKNLS nor RAM show transcriptional activation above empty vector control when co-transfected with CSL, whereas co-transfection of RAMANKNLS with CSL increases transcriptional activity by 24-fold (Figure 2). The addition of both RAM and ANKNLS *in trans* increases CSL-mediated activity by 9-fold. The stronger activation when RAM and ANKNLS are *in cis* (i.e., RAMANKNLS) compared to when RAM and ANKNLS are *in trans* suggests that the whole is greater than the sum of its parts. This observation is consistent with a model in which direct linkage of RAM with ANKNLS (*in cis*) confers an increase in effective concentration of ANK near CTD, thus promoting this weak interaction. In this format, the effective concentration appears to increase transcription by approximately 2.5-fold (9- vs. 24-fold stimulation).

### Effective concentration enhancement by covalent coupling of ANK to CSL

Comparison of the activity of RAM and ANK *in cis* versus *in trans* shows the importance of covalency in transcriptional activation, and is consistent with an enhanced reactivity through increasing the effective concentration of ANK around CSL. Another means to test this effective concentration model is to fuse ANK to the C-terminus of CSL. In the crystal structure of the worm ternary complex [17], the C-terminus of CSL and the N-terminus of ANK are separated by a distance of 9 Å (Figure 1B). To span this distance, we used a spacer of 5-glycyl residues between CSL and ANKNLS, in an effort to promote the binding reaction between ANK and CTD in the absence of RAM. If the sole mechanism by which the bivalent structure of NICD enhances transcriptional activation is effective concentration enhancement, then the CAN (CSL-ANKNLS) fusion should be maximally active in the absence of RAM. Moreover, by separately adding RAM to the CAN fusion, we can test for additional roles of RAM, namely, RAM-induced allostery and/or displacement of co-repressor proteins.

**Figure 3:**
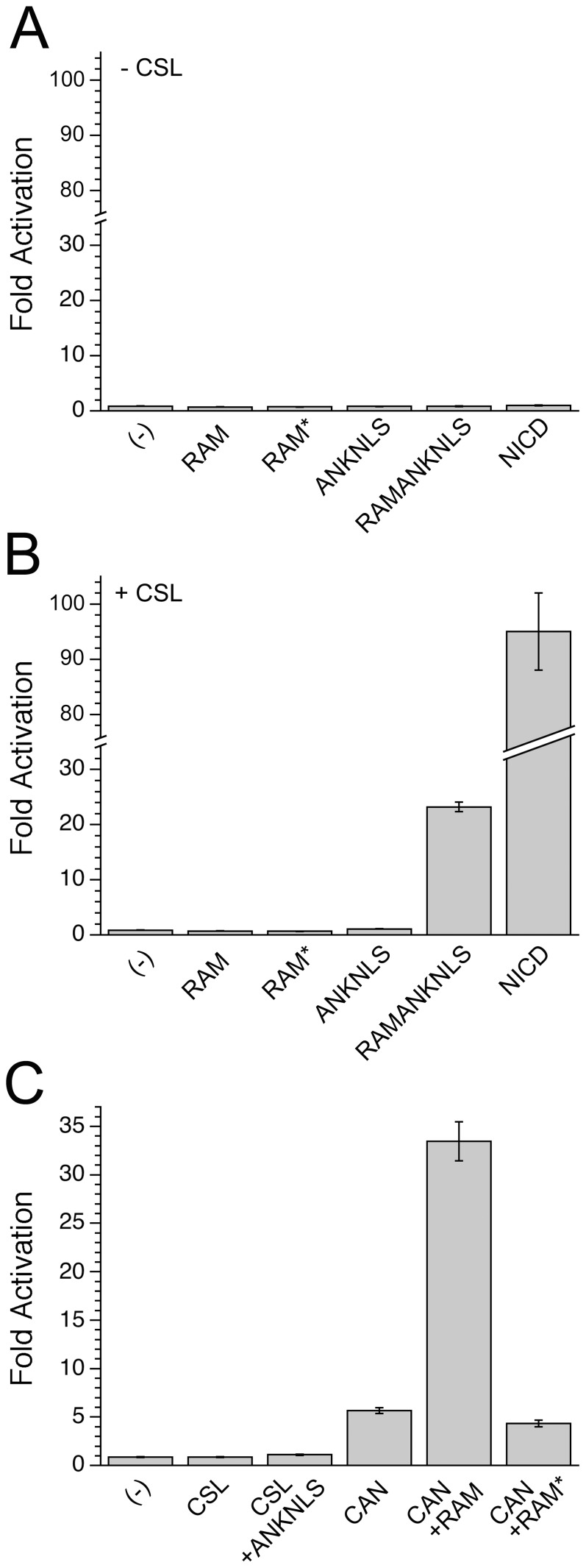
CSL-dependent transcription assays in OT11 allow determination of minimal requirements for ternary complex formation. A). In the absence of CSL, neither full-length NICD nor fragments of NICD can activate transcription. B). In the presence of CSL, only RAMANKNLS and NICD result in transcriptional activation. C). Fusion of CSL-ANKNLS (CAN) activates transcription 6-fold over empty vector alone (and relative to CSL and ANKNLS), consistent with an increased effective concentration of ANK at the CTD of CSL. Co-transfection of RAM with CAN further increases transcriptional activation, implicating an additional role of RAM in forming the active ternary complex.

To examine whether covalency promotes activation by localizing ANK to CSL, we transfected a plasmid encoding our CAN fusion protein into OT11 mouse cells. This cell line lacks endogenous CSL, allowing us to test the transcriptional activity of our CAN fusion protein, and compare these data with the same protein domains in their native connectivities. No activity is detected for cells transfected with CSL alone, confirming the absence of background levels of Notch signaling in OT11 (Figure 3A). Similarly, no activity is detected for cells transfected with either RAMANKNLS or full-length NICD alone, confirming the absence of endogenous CSL in OT11. Co-transfection of CSL and RAMANKNLS displays 23-fold activation (compared to 100-fold activation for cells co-transfected with CSL and full length NICD; Figure 3B). However, no activity is detected for co-transfection of CSL and RAM, or CSL and ANKNLS, consistent with the hypothesis that for mammalian Notch, the ANK:CTD interaction is dependent upon the increased effective concentration of ANK conferred by being tethered to RAM (*in cis*).

Our CAN fusion protein should promote assembly of the CTD:ANK:MAML complex by facilitating the interaction of ANK with CSL. Assuming the fused ANK domain can properly engage both CTD and MAML, this fusion should act as an on-state mimic by being poised to bind the co-activator MAML. Consistent with this prediction, the CAN fusion displays significant activation above both empty vector control, and co-transfection of CSL and ANKNLS *in trans* (Figure 3C). Co-transfection of RAM with CAN further increases the transcriptional activity another 6-fold over CAN alone, slightly exceeding the activity level for the co-transfection of RAMANKNLS with CSL (Figure 3B), the latter having RAM and ANK *in cis* to each other, but *in trans* to CSL. In contrast, co-transfection of CAN and the CSL-binding-incompetent RAM* does not show any increase in activation above CAN alone (Figure 3C). This observation is consistent with the idea that the RAM-linker arrangement in NICD serves both to localize ANK to CSL, thereby increasing its effective concentration, and to further activate the CSL complex.

### Inhibiting CAN-mediated activation by addition of dominant-negative MAML

**Figure 4:**
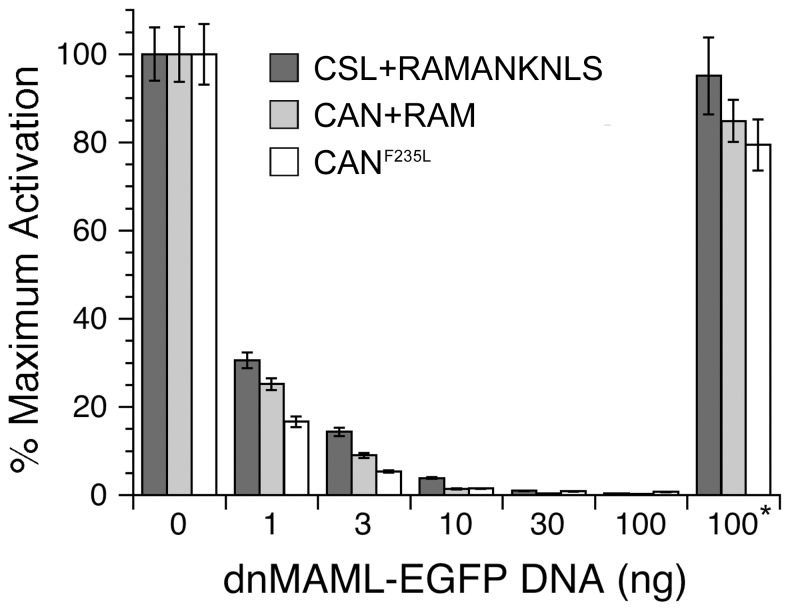
The CAN fusion molecule is fully competent for MAML interaction, suggesting that CAN maintains a native-like CTD:ANK interface. The interaction of the co-activator MAML with the CTD:ANK interface was assessed using dominant-negative MAML (dnMAML) to inhibit transcription. The CAN fusion molecule, in the presence of RAM (light gray), is equally susceptible to dnMAML as CSL and RAMANKNLS *in trans* (dark gray), suggesting that the ANK:CTD interface is formed similarly in both arrangements. The CAN^F235L^ fusion molecule, in the absence of RAM (white), is equally susceptible to dnMAML as both arrangements described above. EGFP alone (*) does not significantly perturb CSL/CAN-mediated transcriptional activation.

To test whether the ANK:CTD interface in our CAN fusion has the same reactivity for downstream activators as that produced by native bivalent association (CSL:RAMANKNLS), we co-transfected a dominant-negative form of MAML (dnMAML) which binds directly to the CSL:ANK interface, thereby disrupting Notch transcriptional activation through competition with full-length MAML [17], [18], [36]–[40]. At the lowest levels of dnMAML tested (1 ng, Figure 4), transcriptional activity is reduced both for CSL co-transfected with RAMANKNLS and for CAN co-transfected with RAM (30 and 25% of maximal [i.e., uninhibited] activation, respectively). At higher dosages of dnMAML, both complexes display similarly reduced output, reaching nearly 0% output at 30 ng dnMAML (Figure 4). Co-transfection of 100 ng EGFP control vector had no effect on CSL/CAN-dependent transcriptional activity (denoted by *, Figure 4). These data suggest that the MAML-binding site (namely, the CSL:ANK interface) is intact in the CAN fusion and is structurally similar to that found between CSL and the ANK domain of bivalent NICD.

### The use of the BTD F235L substitution to disrupt co-repressor binding

In both the experiments testing covalency of RAMANK (Figure 2B) and CSL-ANK (CAN, Figure 3C), we see an effect of RAM that is independent of effective concentration enhancement. RAM increases activity over ANK alone in a *trans* configuration, where effective concentration effects are eliminated. In CAN-expressing cells, where effective concentration should be saturated, RAM further stimulates activation. In both cases, this stimulation involves direct RAM binding to BTD, since RAM* constructs result in no further stimulation. These data do not reveal whether RAM is acting simply by displacing co-repressor proteins, or whether it is enhancing ternary complex formation through allosteric coupling.

To resolve the apparent requirement for RAM in the context of CAN, we assessed the effects of an F235L point substitution within the BTD of CSL. In the context of a triple-alanyl substituted CSL (EEF233AAA), this substitution has been shown to block binding of the co-repressor CIR to BTD, and also perturbs RAM binding [30], [41]. We have previously shown that F235L substitution weakens (but does not abrogate) RAM binding to BTD, without altering structure or stability [21], [42]. To the extent that RAM can interact with CSL^F235L^, we can resolve co-repressor effects from possible allosteric effects.

**Figure 5:**
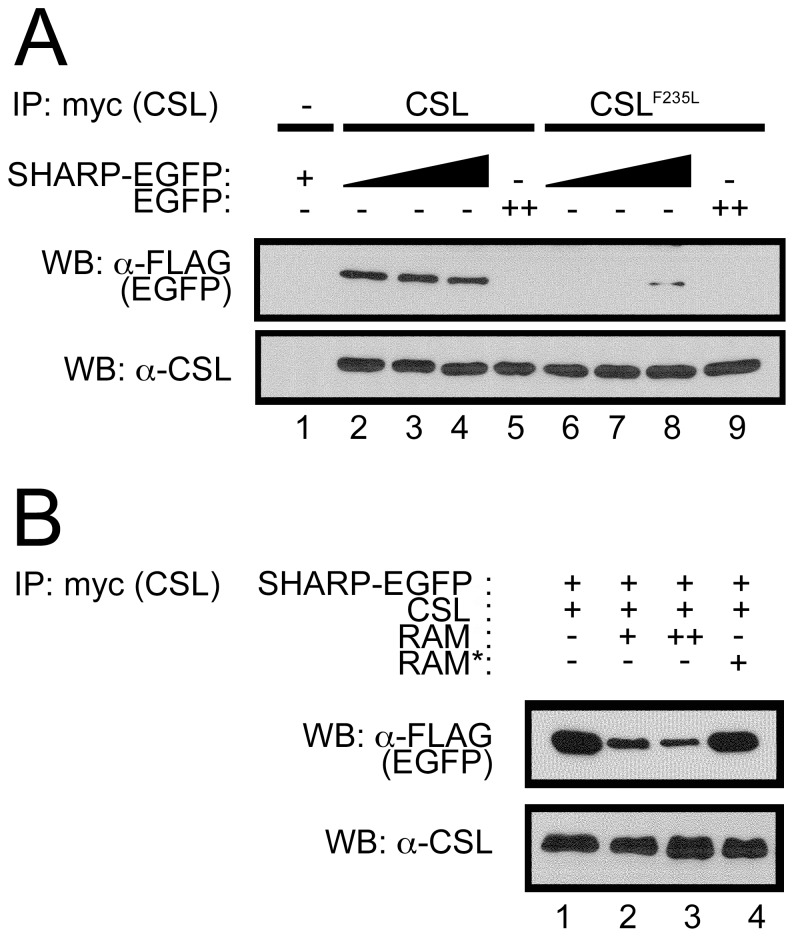
CSL^F235L^ and RAM both perturb the interaction with the co-repressor SHARP. A). Immunoprecipitation of the co-repressor SHARP with CSL, and disruption by F235L point substitution. After co-transfection of HeLa cells with SHARP-EGFP (3xFLAG) and CSL (myc), SHARP-EGFP is readily detected by immunoprecipitation of wild-type CSL (lanes 2–4); no interaction is detected between CSL and EGFP-3xFLAG (lane 5). SHARP-EGFP is only weakly detected at the highest concentration after immunoprecipitation of CSL^F235L^ (lanes 6–8); no interaction is detected between CSL^F235L^ and EGFP-3xFLAG (lane 9), directly demonstrating that the F235L point substitution perturbs co-repressor binding. B). Disruption of SHARP:CSL immunoprecipitation with binding competent RAM. The interaction between CSL and SHARP is perturbed with increasing amounts of RAM peptide, but is unaffected by RAM* peptide (CSL-binding incompetent peptide), demonstrating that RAM directly displaces co-repressor protein(s).

To directly test the effects of the F235L substitution on co-repressor binding, we co-immunoprecipitated the co-repressor protein SHARP (MINT/SPEN) with CSL and CSL^F235L^ (after several failed attempts to detect any interaction between wild-type CSL and CIR). SHARP was readily detected over a range of concentrations after immunoprecipitation of wild-type CSL; no interaction was detected between CSL and EGFP (lanes 2–5, Figure 5A). SHARP was weakly detected only at the highest concentration after immunoprecipitation of CSL^F235L^; again, no interaction was detected between CSL^F235L^ and EGFP (lanes 6–9, Figure 5A). These data demonstrate that the F235L point substitution significantly weakens binding of the co-repressor SHARP. To confirm that RAM can displace this co-repressor, we tested the ability of RAM peptide to directly disrupt SHARP binding to CSL. Addition of RAM peptide disrupts the CSL:SHARP complex in a dose-dependent manner (Figure 5B). In contrast, SHARP:CSL interaction is unperturbed by RAM* (CSL-binding incompetent) peptide. Together, these data show that the displacement of the co-repressor SHARP by RAM can be mimicked by F235L substitution.

### F235L point substitution renders CAN maximally active but refractory to RAM

**Figure 6:**
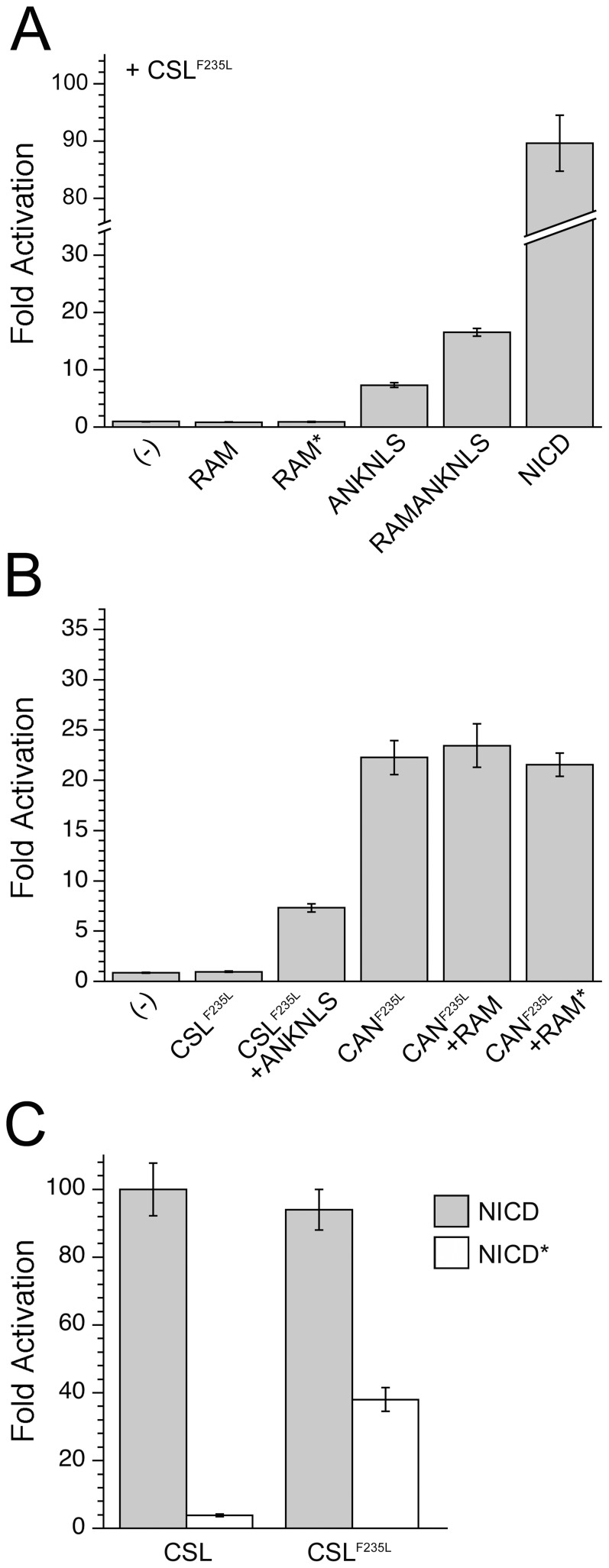
A Phe-to-Leu point-substitution fully activates the CAN fusion. A). In the context of CSL^F235L^, ANKNLS displays an increased level of transcriptional activity, though RAMANKNLS and NICD are largely unchanged, compared to wild-type CSL. B). Fusion of CSL^F235L^ to ANKNLS (CAN^F235L^) increases transcriptional activity by 3-fold compared to the co-transfection of CSL^F235L^ and ANKNLS *in trans*, and renders the CAN^F235L^ construct insensitive to further activation by RAM, unlike CAN (Figure 3C). This observation is consistent with the co-repressor perturbing effects of the F235L point substitution. C). The xWxP motif of RAM maintains interactions with F235L-substituted CSL. Mutation of the xWxP motif of RAM in full-length NICD (denoted NICD*) decreases transcriptional activity with co-transfected CSL from 100- to 4-fold. Mutation of the xWxP in full-length NICD decreases transcriptional activity with co-transfected CSL^F235L^ from 94- to 38-fold, consistent with some level of interaction between the xWxP motif and CSL^F235L^. The increased output of NICD* in the presence of CSL^F235L^ compared to wild-type CSL (38- vs. 4-fold, respectively) is presumably due to diminished co-repressor binding to CSL^F235L^.

The above experiments indicate that RAM both increases the effective concentration of ANK for CSL, and displaces the SHARP co-repressor from CSL. To test whether RAM also activates transcription by inducing allosteric changes within CSL, we introduced the F235L point substitution into our CSL and CAN constructs and monitored the effects on transcriptional activation. Because the F235L substitution is distant from the ANK/MAML binding site, we expect the CAN^F235L^ fusion protein to promote CTD:ANK:MAML interaction to the same extent as the wild-type CAN fusion, but should act as a stronger on-state mimic since the F235L point substitution disrupts co-repressor binding. Consistent with these expectations, the CAN^F235L^ fusion shows an increased level of activation (22-fold above empty vector control) compared to 7-fold for CSL^F235L^ and ANKNLS *in trans*, and 6-fold for the CAN fusion without the F235L point substitution (Figure 6A, B). Significantly, the level of transcriptional activation by CAN^F235L^ is nearly identical to that obtained when RAMANKNLS is co-transfected with CSL. Thus CAN^F235L^ is active at wild-type levels. If RAM were enhancing transcription solely by inducing allosteric changes, we would not expect F235L substitution to enhance activation by CAN, since it seems highly unlikely that this substitution would trigger the same allosteric response. In addition, co-transfection of CAN^F235L^ and RAM does not increase the transcriptional activity compared to CAN^F235L^ (Figure 6B). Although the decrease in affinity for RAM resulting from F235L substitution may also be expected to diminish the RAM response of CAN^F235L^ (see below), when this observation is taken together with the wild-type level of activation seen from CAN^F235L^, these data indicate that in the context of mammalian Notch signaling, RAM contributes to CSL-mediated activation by enhancing effective concentration of ANK and by displacing co-repressor proteins. We find no evidence for activation through allosteric rearrangement of CSL.

### F235L-substituted CSL remains sensitive to the RAM xWxP motif

The effects of the F235L point substitution on transcriptional activation are consistent with a perturbation of co-repressor protein binding to BTD. However, we previously showed that the F235L substitution decreases the affinity of RAM for BTD by 100-fold, although it does not completely eliminate binding [21]. To confirm that RAM can still interact with CSL^F235L^ in transcription assays, we monitored the transcriptional activation of wild-type and xWxP (to A_4_) substituted NICD (denoted NICD*) with wild-type and F235L substituted CSL. Co-transfection of OT11 cells with CSL and NICD yields 100-fold transcriptional activation, whereas co-transfection of CSL and NICD* displays only 4-fold activation. This decrease defines the severity of the full loss of the RAM:BTD interaction on transcriptional activation in this assay. Importantly, CSL^F235L^ remains sensitive to this strongly disruptive binding substitution in RAM: co-transfection of CSL^F235L^ and NICD* diminishes transcriptional activation from that of wild-type NICD to CSL^F235L^ from 94-fold to 38-fold activation (Figure 6C). This 2.5-fold decrease in transcriptional activation upon xWxP to A_4_ substitution suggests that the xWxP motif of RAM maintains a functionally significant interaction with the F235L substituted BTD. In the context of NICD*, the increase in output of F235L-substituted versus wild-type CSL (38-fold compared to 4-fold, respectively) likely results from the resistance to co-repressor binding conferred by the F235L point substitution, but the near wild-type levels of transcriptional activation of wild-type NICD on CSL^F235L^ indicates that RAM can contribute to transcriptional output despite F235L substitution.

### Discussion

The Notch-dependent switch from transcriptional repression to activation is mediated by the DNA-binding transcription factor CSL, and in making this conversion, CSL must shed co-repressor proteins in exchange for co-activators. It has been proposed that displacement of co-repressor proteins is coupled to the high-affinity BTD:RAM binding reaction [29], [41], [43], [44]. In addition, it has been proposed that because of the bivalent structure of the CSL:NICD interaction, RAM-binding leads to an increased effective concentration of ANK at its binding site on CTD [16], facilitating the formation of an active ternary complex by association with MAML [15], [17], [18]. To test these proposals, we constructed a CSL-ANK fusion protein that should increase the effective concentration of ANK at CTD without RAM binding. Moreover, by adding RAM to this CAN fusion, we can examine additional roles of RAM in transcriptional activation independent of changes in effective concentration. By combining these studies with a CSL variant that has weakened interactions with co-repressors, we can explore whether these additional effects result from co-repressor displacement or RAM-induced allosteric changes.

### RAM promotes ANK:CTD binding when *in cis*

Of the ∼100 residues of NICD preceding the ANK domain, the N-terminal 20 residues (centered on the xWxP motif) are responsible for high-affinity binding to the BTD [19], yet the functional relevance of the intervening 80 residues of the linker has yet to be determined. By dissecting NICD into two separate regions (RAM and ANKNLS *in trans*), we are able to evaluate the transcriptional enhancement conferred by the linking of RAM and ANK. When RAM and ANK are separate, neither RAM nor ANK shows any transcriptional activation, although co-transfection of both RAM and ANK *in trans* results in partial activation (9-fold compared to 25-fold when RAM and ANK are *in cis*). These data confirm that RAMANK is a more potent activator than the same domains *in trans* (by a factor of 3), consistent with previously published results from both vertebrates and invertebrates [11], [13]. We interpret this 3-fold enhancement as the concentration enhancement of ANK in the wild-type *cis* configuration.

### CSL-ANK “On-State” Mimicry

Based on both the hypothesis that RAM increases the interaction between CTD and ANK by localization, and the structural insights provided by crystallographic studies of the Notch ternary complex [17], [18], we made a CSL-ANKNLS (CAN) fusion molecule. To the extent that RAM enhances Notch activity by providing an increased local concentration of ANK near CSL, this fusion should act as an on-state mimic, circumventing this requirement of RAM. Indeed, the CAN fusion displays a significant level of transcriptional activity. This particular role of RAM has been debated to be non-essential in the past, as mixtures of purified CSL and MAML can, in the absence of co-repressor proteins, form a ternary complex *in vitro* (albeit inefficiently, requiring an overwhelming excess of ANK) [12], [14], [15], [45], [46].

However, wild-type levels of transcription are only achieved after the addition of RAM *in trans*, suggesting another role for RAM in transcriptional activation, either to displace co-repressor proteins or to induce an activating conformational change in CSL. To help resolve this, we introduced the F235L point substitution into CSL, which we have shown to displace the co-repressor SHARP from CSL (Figure 5A). This substitution increases the transcriptional activity of the CAN fusion, approaching that of the wild-type connectivity (22-fold for CAN^F235L^, 25-fold for CSL and RAMANKNLS). This observation supports the role of co-repressors in limiting the activity of CAN compared to CSL and RAMANKNLS (6-fold versus 25-fold), and supports the interpretation that RAM further activates CAN (Figure 3C) by co-repressor displacement. It is remarkable that removal of only half a phenyl ring can result in such a large increase in activation and a large decrease in co-repressor binding.

Ideally, the interpretation that RAM activates mammalian CAN through displacement of co-repressors rather than by allosteric changes in CSL could be directly tested by assessing whether the RAM enhancement is ameliorated in a CSL variant that fails to bind co-repressors. Although the insensitivity of CAN^F235L^ to the addition of RAM is consistent with the co-repressor displacement mechanism, this test is imperfect, since the F235L substitution also decreases the affinity of CSL for RAM [21]. Thus, part of this insensitivity could be due to a decreased affinity of RAM for this point substitution. However, the observation that alanine substitution of the NICD xWxP motif decreases transcriptional activity in CSL^F235L^ (Figure 6C) indicates that the xWxP motif can still engage its site on F235L-substituted BTD.

The observation that combined fusion of ANKNLS to CSL and F235L substitution to BTD can account for full transcriptional activity of RAMANKNLS suggests that long-range allosteric activation by RAM is not required to reach maximal transcriptional output in the context of the mammalian homologues. Friedmann *et* *al*., 2008 suggested that in *C. elegans*, a RAM-induced NTD loop rearrangement facilitates formation of the active ternary complex *in vitro* based on synergistic effects in gel-shift assays, but saw reduced synergism with mammalian homologues. The results presented here using mammalian cell culture indicate that full activation can be achieved without RAM binding. While our data cannot definitively rule out the possible effects of subtle RAM-induced conformational changes in CSL, the differences in results between *C. elegans* [20] and mammals (this study) suggests a lack of mechanistic conservation amongst divergent species, namely worm and human. This divergence in mechanisms is consistent with an observation that in *C. elegans*, ANK alone is sufficient to induce transcriptional activation in the absence of RAM [13], [14]. It is possible that co-repressor proteins play a smaller role in limiting CSL-mediated transcription in *C. elegans*. Alternatively, the recent findings that two co-repressors use domains of CSL outside the BTD [23], [35] suggest the possibility that the ANK domain may directly displace co-repressors, and that perhaps in worm, the co-repressors tend to interact outside of BTD.

The consequences of fusing CSL and portions of NICD have been examined previously, although the results varied. Kurooka *et* *al*., 1998 made a CSL-ANK fusion using mouse proteins and did not observe any transcriptional activation, but did observe transcriptional activation when CSL was fused to full-length NICD (lacking only the RAM region). Wettstein *et* *al*., 1997 also made a CSL-ANK fusion using frog proteins, and observed a low level of transcriptional activation. Neither of these studies examined the consequences of adding RAM *in trans* to their CSL-ANK fusion proteins. The study here differs from these earlier studies in that we fused the CTD of CSL and ANK domain of NICD in close proximity based on crystal structures of CSL:NICD complexes [17], [18], whereas the previous studies were based on the minimal domains required for transcriptional activation. We also examine the roles of F235L point substitution and RAM in directly displacing the co-repressor SHARP from CSL.

### RAM-ANK bivalency as a means to suppress intermediate states in Notch activation

**Figure 7:**
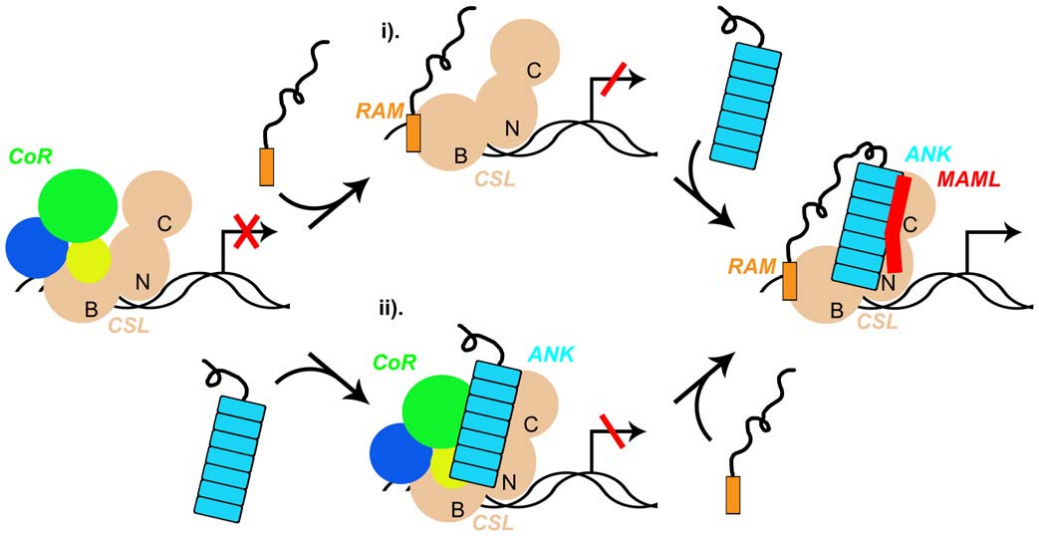
The coupling of RAM and ANK *in cis* decreases the probability of forming transcriptional intermediates. If the RAM and ANK domains of NICD bound CSL independently *in trans*, the CSL switch from repression to transcriptional activation could be compromised by intermediate states of ligation, with either i) RAM bound to BTD without the ANK:CTD interaction intact, or ii) ANK bound to CTD without the BTD:RAM interaction intact. These intermediary states would result in either relief of repression without activation, or simultaneous repression and activation, respectively. This model predicts that only when RAM and ANK are coupled (*in cis*) does Notch signaling make a sharp transition from complete repression to full transcriptional activation.

The CAN fusion protein provides access to intermediates along the Notch pathway that would otherwise be difficult to probe. If the RAM and ANK domains bound independently *in trans*, the switch from repression to activation presumably could populate partly bound intermediates states: i.) CSL with RAM bound to BTD but without bound ANK, and ii.) CSL with ANK/MAML bound to CTD but without bound RAM (Figure 7). Partly ligated state i.) would have displaced the co-repressors (i.e., SHARP) that are in direct competition with RAM binding, but would not bind MAML. Partly ligated state ii.) would bind ANK/MAML, albeit inefficiently, due to the presence of co-repressor proteins interacting with both BTD and CTD. Thus, the first type of intermediate is in a state that is neither repressed nor activated, whereas the second type of intermediate is both repressed and activated. The coupling of RAM and ANK *in cis* decreases the probability that these intermediates will form, as the high-affinity binding of RAM to BTD displaces co-repressor proteins from BTD while simultaneously promoting the CTD:ANK interaction necessary to achieve full transcriptional activation. This should promote a sharp switch from the repressed to the activated state. As the levels of active Notch are tightly regulated, with disease states such as cancer and tissue malformations resulting from too much or too little Notch signaling, minimizing the population of intermediates may be important to precisely control the level of activation.

### Conclusions

We present data revealing how the covalency of RAM-ANK can effectively suppress intermediates of Notch signaling by coupling the actions of co-repressor displacement by RAM and concentration enhancement of ANK. We also present an engineered transcription factor that, through the use of a fusion and a single point substitution, can act as an on-state mimic of the Notch ternary complex, producing nearly wild-type levels of activation. This high level of activation is achieved in the absence of RAM, the strongest interacting segment of NICD. As over-active Notch signaling has been implicated in over 60% of all childhood cases of T-cell acute lymphoblastic leukemia (T-ALL) [47] and much research is dedicated to the design of therapeutics targeting the active ternary complex [48], the CAN^F235L^ fusion protein presented here could serve as a simple cell-culture-based model of T-ALL, allowing for high-throughput screening of potential therapeutic agents.

## Materials and Methods

### Subcloning

The CSL construct studied here contains residues 10–436 of human isoform 1 subcloned into the pcDNA3.1-myc/his vector backbone, as previously described [21]. The CAN fusion protein contains human CSL as described above, followed by a five-glycyl linker, and human Notch1 residues 1857–2174, corresponding to the 0^th^ through 7^th^ ankyrin repeats and the bi-partite nuclear localization sequence. The F235L point substitution was introduced into CSL and CAN constructs *via* QuikChange® mutagenesis, giving rise to CSL^F235L^ and CAN^F235L^, respectively. RAMANKNLS, ANKNLS, and RAM were all subcloned from hNICD1 into a pcDNA3.1-myc/his vector, corresponding to residues 1758–2174, 1857–2174, and 1758–1878, respectively. NICD* and RAM* have the xWxP motif (residues 1767–1770) substituted with four alanines (A_4_), rendering them unable to bind to the BTD of CSL [21]. For the co-immunoprecipitation experiments with co-repressor proteins, enhanced green fluorescent protein (EGFP) was subcloned into a modified pcDNA vector with a C-terminal 3xFLAG® tag, and a PCR amplified synthetic gene encoding residues 2801–2862 of the human SPEN homologue SHARP was inserted in between EGFP and the 3xFLAG® tag, yielding a EGFP-SHARP-3xFLAG® fusion protein.

### Reporter Assays

OT11 (CSL^−/−^) cells were a generous gift from Tasuku Honjo (Kyoto University) [44] and were maintained at 37°C in Dulbecco's Modified Eagle Medium (DMEM) supplemented with 10% (v/v) fetal bovine serum (FBS) and 1% (v/v) penicillin/streptomycin. Cells were seeded in 24-well plates at a density of 1×10^5^ cells per well, 24 hours prior to transfection with Lipofectamine LTX (Invitrogen) as per the manufacturer's instructions. Each well was transfected with 225 ng TP1-luc reporter plasmid (firefly luciferase under the control of a 10x CSL binding site promotor) [12], 75 ng Renilla tranfection control plasmid, 0–200 ng experimental plasmid, and 200–0 ng empty pcDNA vector to maintain a constant 500 ng total DNA per well. For the CAN experiments testing the effects of covalency, 100 ng of each domain-expressing plasmid was transfected. Cells were harvested 40–44 hrs post-transfection, lysed in Passive Lysis Buffer, and assayed using the Dual-luciferase Reagent on a GloMax Multi microplate luminometer per the manufacturer's instructions (Promega). Each experiment was done in quadruplicate and repeated at least three times, with the average +/− standard error reported in Table S1.

### Co-Immunoprecipiation

HeLa cells (ATCC #CCL-2; Manassas, VA) were maintained at 37°C in DMEM supplemented with 10% (v/v) FBS and 1% (v/v) penicillin/streptomycin. Cells were seeded in 6-well plates at a density of 5×10^5^ cells per well. After 24 hours, cells were transfected with 2500 ng/well of CSL, CSL^F235L^, EGFP-SHARP, or EGFP alone. After 48 hours, cells were lysed in cold co-IP buffer (150 mM NaCl, 20 mM NaPO_4_^−^ pH 7.4, 1 mM EDTA (pH 8.0), 1% Triton-X 100, 1 EDTA-free protease inhibitor tablet per 50 mLs) and centrifuged for 20 minutes at 21,000×g to clear cell debris. Reactions were incubated at 4°C for 2 hours before adding 1.5 µg of anti-myc antibody (9E10, Sigma-Aldrich), mixed at 4°C for 2 hours before adding 30 µL Protein G magnetic beads (New England Biolabs, Mass.), and mixed at 4°C for 2 hours. Precipitated beads were washed three times with cold co-IP buffer, resuspended in 50 µL 2x Laemmli buffer, and stored at −80°C. The amount of CSL was kept constant across all experimental conditions, with increasing amounts of EGFP-SHARP (1×, 2×, 4×), or EGFP only (4×). In a separate experiment, the relative amounts of CSL and EGFP-SHARP were kept constant across all experimental conditions, and were treated with increasing amounts of RAM peptide (10 and 20 µM), or RAM* peptide (20 µM) during the 2 hr incubation period immediately preceding addition of primary antibody. Protein levels were detected using Western blot analysis with the following commercially available antibodies: anti-myc (9E10, Sigma-Aldrich), anti-FLAG-HRP conjugate (ab49763, Abcam), anti-myc-HRP conjugate (9B11, #2040, Cell Signaling), anti-CSL (sc-55019, Santa Cruz), and donkey anti-goat-HRP (sc-2020, Santa Cruz).

## Supporting Information

Figure S1 Potential RAM-induced conformational changes in CSL. A). Structural alignment of worm CSL in the absence (deep purple) and presence (wheat) of RAM (orange) reveals two distinct regions of conformational change. One conformational change is proximal to the site of RAM binding (lower right), converting an open loop, lacking regular secondary structure, into a short beta-strand that makes extensive hydrogen bonding with RAM. Phe 235 is represented by a sphere to highlight the significant rearrangement coupled to RAM binding. A second, much more distant conformational change involves a loop rearrangement in the N-terminal domain (NTD). Asp 88 is represented by a sphere. B). The NTD-loop rearrangement is presumably required to bind MAML (red) without steric clash, as modeled here by structural alignment of worm CSL:DNA, RAM:CSL:DNA, and RAMANK:CSL:MAML:DNA, PDB codes 1TTU (apo, purple), 3BRD (holo, wheat), and 2FO1 (MAML, red), respectively.
(TIF)

Table S1 Fold activation and standard error for CSL-dependent transcription reporter assay. Each reaction includes 225 ng TP1-luc reporter plasmid and 75 ng *Renilla* transfection-control plasmid DNA in addition to those components listed in the table.
(PDF)
