# Supplementary figures and images for: Dissecting and Circumventing the Requirement for RAM in CSL-Dependent Notch Signaling
Source: PLoS One. 2012 Aug 2;7(8):e39093. doi: 10.1371/journal.pone.0039093 (PMC3410904; doi:10.1371/journal.pone.0039093)

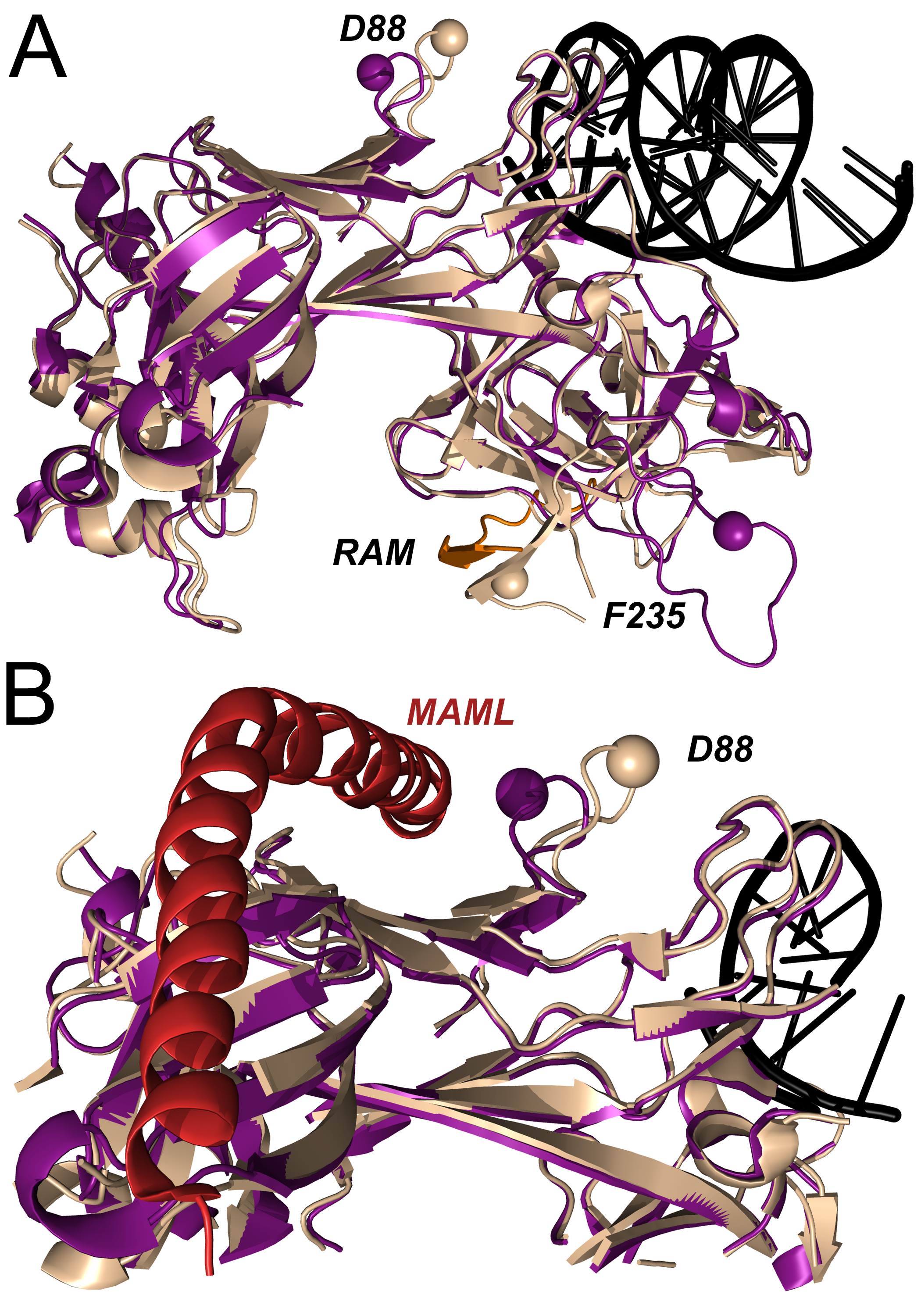

Supplement: Figure S1 — Potential RAM-induced conformational changes in CSL. A). Structural alignment of worm CSL in the absence (deep purple) and presence (wheat) of RAM (orange) reveals two distinct regions of conformational change. One conformational change is proximal to the site of RAM binding (lower right), converting an open loop, lacking regular secondary structure, into a short beta-strand that makes extensive hydrogen bonding with RAM. Phe 235 is represented by a sphere to highlight the significant rearrangement coupled to RAM binding. A second, much more distant conformational change involves a loop rearrangement in the N-terminal domain (NTD). Asp 88 is represented by a sphere. B). The NTD-loop rearrangement is presumably required to bind MAML (red) without steric clash, as modeled here by structural alignment of worm CSL:DNA, RAM:CSL:DNA, and RAMANK:CSL:MAML:DNA, PDB codes 1TTU (apo, purple), 3BRD (holo, wheat), and 2FO1 (MAML, red), respectively. (TIF) [file pone.0039093.s001.tif]
