# Supplementary material for: Dissecting and Circumventing the Requirement for RAM in CSL-Dependent Notch Signaling
Source: PLoS One. 2012 Aug 2;7(8):e39093. doi: 10.1371/journal.pone.0039093 (PMC3410904; doi:10.1371/journal.pone.0039093)
Supplement: Table S1 — Fold activation and standard error for CSL-dependent transcription reporter assay. Each reaction includes 225 ng TP1-luc reporter plasmid and 75 ng Renilla transfection-control plasmid DNA in addition to those components listed in the table. (PDF) [file pone.0039093.s002.pdf]

Component A	Component B	Component C	F. A.	S. E.
(-)	(-)	pcDNA (200ng)	0.88	0.04
(-)	RAM (100ng)	pcDNA (100ng)	0.71	0.03
(-)	RAM* (100ng)	pcDNA (100ng)	0.76	0.04
(-)	ANKNLS (100ng)	pcDNA (100ng)	0.82	0.04
(-)	RAMANKNLS (100ng)	pcDNA (100ng)	0.83	0.04
(-)	NICD (100ng)	pcDNA (100ng)	1.01	0.06
CSL (100ng)	(-)	pcDNA (100ng)	0.87	0.05
CSL (100ng)	RAM (100ng)	(-)	0.70	0.03
CSL (100ng)	RAM* (100ng)	(-)	0.67	0.03
CSL (100ng)	ANKNLS (100ng)	(-)	1.10	0.06
CSL (100ng)	RAMANKNLS (100ng)	(-)	23.1	0.86
CSL (100ng)	NICD (100ng)	(-)	100.0	6.99
(-)	(-)	pcDNA (300ng)	0.23	0.01
CSL (100ng)	(-)	pcDNA (200ng)	0.87	0.05
CSL (100ng)	RAM (100ng)	pcDNA (100ng)	0.70	0.03
CSL (100ng)	RAM* (100ng)	pcDNA (100ng)	0.67	0.03
CSL (100ng)	ANKNLS (100ng)	pcDNA (100ng)	0.58	0.12
CSL (100ng)	RAM (100ng)	ANKNLS (100ng)	9.22	1.61
CSL (100ng)	RAM* (100ng)	ANKNLS (100ng)	1.13	0.18
CSL (100ng)	RAMANKNLS (100ng)	pcDNA (100ng)	24.9	2.02
(-)	(-)	pcDNA (200ng)	0.88	0.04
CSL (100ng)	(-)	pcDNA (100ng)	0.87	0.05
CSL (100ng)	ANKNLS (100ng)	(-)	1.10	0.06
CAN (100ng)	(-)	pcDNA (100ng)	5.66	0.28
CAN (100ng)	RAM (100ng)	(-)	33.4	1.99
CAN (100ng)	RAM* (100ng)	(-)	4.34	0.33
CSL^{F235L} (100ng)	(-)	pcDNA (100ng)	0.96	0.06
CSL^{F235L} (100ng)	RAM (100ng)	(-)	0.85	0.02
CSL^{F235L} (100ng)	RAM* (100ng)	(-)	0.92	0.04
CSL^{F235L} (100ng)	ANKNLS (100ng)	(-)	7.32	0.41
CSL^{F235L} (100ng)	RAMANKNLS (100ng)	(-)	16.5	0.69
CSL^{F235L} (100ng)	NICD (100ng)	(-)	94.5	4.90
(-)	(-)	pcDNA (200ng)	0.88	0.04
CSL^{F235L} (100ng)	(-)	pcDNA (100ng)	0.96	0.06
CSL^{F235L} (100ng)	ANKNLS (100ng)	(-)	7.32	0.41
CAN^{F235L} (100ng)	(-)	pcDNA (100ng)	22.2	1.69
CAN^{F235L} (100ng)	RAM (100ng)	(-)	23.4	2.15
CAN^{F235L} (100ng)	RAM* (100ng)	(-)	21.5	1.15
CSL (100ng)	NICD (100ng)	(-)	100.0	6.99
CSL (100ng)	NICD* (100ng)	(-)	3.84	0.38
CSL^{F235L} (100ng)	NICD (100ng)	(-)	94.5	4.90
CSL^{F235L} (100ng)	NICD* (100ng)	(-)	38.5	3.53
